# Maqui berry cystatin inhibits cathepsin k activity and stimulates osteogenic differentiation of human dental pulp cells

**DOI:** 10.1590/1678-7765-2025-0661

**Published:** 2026-02-16

**Authors:** Luana Raphael da Silva, Eduardo Pereira de Souza, Bárbara Roma Mendes, Breno Henrique Amancio, Flávio Henrique-Silva, Gisele Faria

**Affiliations:** 1 Universidade Estadual Paulista Faculdade de Odontologia de Araraquara Departamento de Odontologia Restauradora Araraquara São Paulo Brasil Universidade Estadual Paulista (UNESP), Faculdade de Odontologia de Araraquara, Departamento de Odontologia Restauradora, Araraquara, São Paulo, Brasil; 2 Universidade Federal de São Carlos Departamento de Genética e Evolução São Carlos São Paulo Brasil Universidade Federal de São Carlos (UFSCar), Departamento de Genética e Evolução, São Carlos, São Paulo, Brasil.

**Keywords:** Cathepsin K, Cystatins, Dental pulp

## Abstract

**Objectives:**

Cystatins, endogenous inhibitors of cysteine proteases, regulate extracellular matrix degradation. Their plant-derived homologs (phytocystatins) include MaquiCPI-3, a recombinant protein obtained from Aristotelia chilensis (maqui berry). This study aimed to investigate the inhibitory effect of MaquiCPI-3 on human cathepsin K (CTSK) activity and its cytotoxicity and impact on the proliferation, migration, and osteogenic differentiation of human dental pulp cells (hDPCs).

**Methodology:**

The inhibitory activity of MaquiCPI-3 against CTSK was measured using a spectrofluorometer with the fluorogenic substrate Z-Phe-Arg-AMC. The hDPCs from third molars were characterized by flow cytometry for mesenchymal (CD90, CD73, CD105) and hematopoietic (CD34, CD45) markers. The hDPCs, either exposed to MaquiCPI-3 or left untreated (control), were assessed for viability (MTT assay), proliferation (bromodeoxyuridine incorporation), chemotaxis (Transwell assay), mineralized nodule formation (Alizarin Red S staining), alkaline phosphatase activity (thymolphthalein release), and expression of mineralization-related genes (qPCR). Data were analyzed using one- or two-way ANOVA with appropriate post hoc tests or nonparametric alternatives (α=0.05).

**Results:**

MaquiCPI-3 potently inhibited CTSK (Ki=1.72 nM, Ki,app=2.08 nM), showed no cytotoxicity, and significantly enhanced ALP activity, mineralized nodule formation, and expression of BMP-2 and osteocalcin, stimulating no hDPC proliferation or migration when compared with the control.

**Conclusions:**

MaquiCPI-3 increased no cell proliferation or migration, its ability to inhibit CTSK activity and induce an osteogenic phenotype shows promising potential therapeutic strategies aimed at repairing and regenerating pulp and periapical tissues and controlling bone resorption.

## Introduction

Bone remodeling is a dynamic process regulated by the balance between osteoclast and osteoblast activity.^1^ When this balance is disrupted and bone resorption prevails, pathological conditions such as osteoporosis, arthritis, periodontal disease, and apical periodontitis may develop.^2,3^ Osteoclasts degrade the mineralized matrix by local acidification and the organic matrix via proteases, including matrix metalloproteinases and cysteine proteases such as cathepsin K (CTSK).^4^

CTSK is the main lysosomal protease expressed by osteoclasts. It is essential for bone resorption.^5^ In addition to degrading type I collagen—the primary component of the bone matrix—and type II collagen,^6^ CTSK regulates osteoclast and macrophage differentiation and function.^7^ In inflammatory diseases such as apical periodontitis, CTSK expression is upregulated and associated with bone resorption and periapical lesion progression.^8^ Adjunctive strategies for conventional endodontic treatment have been investigated to enhance periapical repair. Among these, CTSK inhibition-based therapies to reduce bone resorption have shown promising results.^9^ However, synthetic inhibitors such as Odanacatib were discontinued because of severe systemic adverse effects.^10^

In this context, natural inhibitors of cysteine proteases, such as phytocystatins (a class of plant cystatins), have emerged as potential modulators of cathepsin activity.^11^ Recombinant phytocystatins, including C*sin*CPI-2 from *Citrus sinensis* (sweet orange) and CaneCPI-5 from sugarcane, have shown inhibitory activity against human cathepsins, including CTSK, together with anti-inflammatory and pro-osteogenic effects *in vitro* and *in vivo.*^12-15^

*Aristotelia chilensis* (Mol.) Stuntz, commonly known as maqui berry (often shortened to maqui), is a native South American plant that produces purple fruits (maqui berries, popularly known as Chilean wineberries) that are traditionally used as food and in folk medicine.^16^ Its fruits and leaves have attracted attention because of their high content of bioactive compounds, particularly anthocyanins, which provide antioxidant, anti-inflammatory, cardioprotective, antidiabetic, and wound-healing properties.^17^ A recent study has found six cystatins from this plant, termed MaquiCPI-1 to MaquiCPI-6. Among them, MaquiCPI-3 showed high production yields in the *Escherichia coli* expression system and inhibitory activity against papain and human cathepsins B and L, suggesting therapeutic potential.^18^ Beyond its traditional use, maqui berry has also been explored for biotechnological applications in dentistry; MaquiCPI-3 has shown the ability to prevent initial enamel and dentin demineralization.^18,19^ However, its effect on CTSK remains unknown.

Regenerative endodontics is based on biological principles to restore the dentin–pulp complex and periapical tissues by using substances that stimulate mesenchymal stem cells (MSCs) to differentiate into tissue-forming cells.^20^ The search for bioactive molecules that induce the osteoblastic and/or odontoblastic differentiation of MSCs remains a major challenge in developing therapeutic approaches for pulp and periapical repair and regenerative endodontics.^21^

Considering the osteoinductive potential of phytocystatins in cellular models^13-15^and their inhibitory activity against CTSK,^14^ the aim of this study was to evaluate the ability of MaquiCPI-3 to inhibit CTSK activity and to assess its effects on human dental pulp cells (hDPCs), analyzing its cytotoxicity and its impact on key processes in tissue repair and regeneration, namely migration, proliferation, and osteogenic differentiation. The null hypothesis was that MaquiCPI-3 had no inhibitory effect on CTSK and did not influence the viability, migration, proliferation, or osteogenic differentiation of hDPCs.

## Methodology

### Expression and purification of recombinant MaquiCPI-3 protein

For protein expression, *Escherichia coli* Rosetta cells (transformed with the plasmid pET28aMaquiCPI-3) were used as described by Souza, et al.^18^ (2023). Expression was induced in bacterial cultures with IPTG (isopropyl β-D-1-thiogalactopyranoside), followed by centrifugation and sonication to obtain the soluble fraction. Purification was performed by affinity chromatography using a nickel resin column (Ni-NTA Superflow, Qiagen), and the eluted protein was dialyzed in phosphate-buffered saline (PBS) 1X (137 mM NaCl, 10 mM phosphate, 2.7 mM KCl, pH 8.0) with three exchanges. Protein concentration was determined using the Pierce BCA Protein Assay Kit (Thermo Fisher Scientific), and the purified protein was stored at –20 °C until use.

### Inhibition of human cathepsin K activity

Enzymatic inhibition assays were conducted at 37 °C in quartz cuvettes using 100 mM sodium acetate buffer (pH 5.5) containing 2.5 mM dithiothreitol (DTT). Human CTSK (1.7 nM, Sigma-Aldrich) was pre-incubated for 5 min. Then, the fluorogenic substrate benzyloxycarbonyl-L-phenylalanyl-L-arginine-7-amido-4-methylcoumarin (Z-Phe-Arg-AMC) was added (1.6 µM). Release of 7-amino-4-methylcoumarin (AMC) was monitored over time with a spectrofluorometer (excitation: 380 nm; emission: 440 nm). Increasing concentrations of MaquiCPI-3 were sequentially added, and residual activity (hydrolysis rate) was recorded every minute. All assays were performed in triplicates. The apparent inhibition constant (K_i_,app) was determined based on the slope of the velocity–inhibitor concentration curve, as described by Nagase and Salvesen^22^ (2001).


$$\frac{V_{0}}{V_{i}}=\frac{\left[I\right]}{Ki,app}$$

Where:

V₀ = hydrolysis rate in the absence of inhibitor

Vᵢ = hydrolysis rate in the presence of inhibitor

[I] = inhibitor concentration

K_i_,app = apparent inhibition constant

The K_i_ value was then calculated according to equation (1)^22^, using a Km value of 7.5 μM.^23^


$$Ki=\frac{Ki_{app}}{1+\frac{\left[S\right]}{Km}}$$

Where:

Km = substrate concentration that yields half of the maximum velocity

[S] = substrate concentration

K_i_ = inhibition constant

### Establishment of human dental pulp cell cultures

Pulp tissues were obtained from healthy human third molars of three patients indicated for orthodontic extraction after approval by the Ethics Committee of the School of Dentistry of Araraquara – UNESP (protocol nº 89380025.6.0000.5416) and after participants signed informed consent forms. The teeth were fractured with a hammer and their pulp tissues were extracted and fragmented. Cell dissociation was performed with type I collagenase (3 mg/mL, Gibco/Life Technologies, Grand Island, NY, USA) for 3 h. Cells were cultured in α-minimum essential medium (α-MEM) supplemented with 10% fetal bovine serum (FBS) (Gibco/Life Technologies, Carlsbad, CA, USA), penicillin (100 IU/mL), and streptomycin (100 µg/mL) (Gibco/Life Technologies) in an incubator at 37 °C and 5% CO₂. After reaching 80% confluence, the cells were subcultured and cryopreserved at passage 3. Experiments were conducted with cells from passages 4 to 6. Cells from the three donors were used in pooled form. Each experimental condition was performed from quadruplicate to sextuplicate (technical replicates) and repeated twice (biological replicates) given the minimal variability between independent assays. Washout lasted for at least 5–7 days depending on the assay.

### Immunophenotypic characterization of hDPCs

At passage 4, the hDPCs cultured in 100 mm Petri dishes (Corning Inc., Corning, NY, USA) (~90% confluence) were dissociated with a non-enzymatic cell dissociation solution (Gibco, Thermo Fisher Scientific, Waltham, MA, USA), centrifuged, and resuspended in stain buffer (2% bovine serum albumin [BSA] in PBS) (Sigma-Aldrich, St. Louis, MO, USA). For phenotypic analysis, 5×10⁵ cells were incubated with phycoerythrin (PE)- or fluorescein isothiocyanate (FITC)-conjugated monoclonal antibodies targeting MSC markers (CD90, CD73, CD105) and hematopoietic/endothelial markers (CD34, CD45) (BD Biosciences FACS Verse 4C, Pharmingen, San Jose, CA, USA). After washing, the cells were analyzed by flow cytometry (CytoFLEX System B5-R3-V5; Beckman Coulter, Brea, CA, USA) operated by CytExpert 2.3 (Beckman Coulter, Brea, CA, USA).^24^

### Cell Viability/Metabolism – MTT Assay

The cell viability of hDPCs treated with MaquiCPI-3 was assessed using the colorimetric MTT assay (3-[4,5-dimethylthiazol-2-yl]-2,5-diphenyltetrazolium bromide) in 96-well plates (Corning). The cells were seeded at 1.2×10⁴ cells/well for the 24-h assay and 0.33×10⁴ cells/well for the 17-day assay. For the 24-h assay, cells were treated with MaquiCPI-3 at concentrations of 0.025, 0.05, 0.1, 0.2, and 0.4 µg/µL together with the control (α-MEM + 10% FBS). For the 17-day assay, concentrations of 0.025, 0.05, and 0.1 µg/µL were tested, and the control consisted of α-MEM + 10% FBS supplemented with osteogenic factors (0.2 mM L-ascorbic acid [A8960, Sigma-Aldrich] and 4 mM β-glycerophosphate [G9422, Sigma-Aldrich]). After incubation, an MTT solution (0.5 mg/mL) was added, and the cells were incubated for 3 h. Formazan crystals were solubilized in isopropanol, and absorbance was measured at 570 nm. Values were normalized to controls, and succinate dehydrogenase activity was used as an indicator of cellular metabolism. Experiments were performed in sextuplicate and repeated twice. The 17-day assay was conducted in parallel with alizarin red staining to monitor cell viability.

### Alkaline Phosphatase (ALP) Activity

The hDPCs were seeded in 96-well plates (0.33×10⁴ cells/well) and incubated for 24 h to enable adhesion. They were then treated with MaquiCPI-3 at concentrations of 0.025, 0.05, and 0.1 µg/µL and controls (α-MEM + 10% FBS, with or without osteogenic supplementation) for 14 days, with medium exchanges every 48 h. The cells were lysed with 0.1% sodium lauryl sulfate (1 mg/mL) for 40 min at room temperature. ALP activity was determined from 50 µL aliquots of lysates using a commercial kit (Labtest, Lagoa Santa, MG, Brazil) according to the manufacturer’s instructions. Absorbance was measured at 590 nm, and results were shown as µmol thymolphthalein/min/L/OD. Activity was normalized to total protein content, quantified with a specific kit (Labtest). Experiments were performed in sextuplicate and repeated twice. MaquiCPI-3 at 0.05 µg/µL was selected for RT-qPCR and chemotaxis assays since this concentration induced the highest ALP activity.

### Alizarin Red Staining (ARS)

Calcium deposition was evaluated by alizarin red staining (ARS). HDPCs were seeded (1.7 × 10⁴ cells/well) in 24-well plates and exposed to MaquiCPI-3 at 0.025, 0.05, and 0.1 µg/µL and controls (osteogenic and non-osteogenic media) for 17 days, with medium changes every 48 h. At the end of the period, the cells were fixed in 70% ethanol (4°C) for 1 h, washed with PBS, and stained with 40 mM alizarin red solution (pH 4.3) for 20 min at room temperature. Excess dye was removed with deionized water washes, and, after air drying, images were captured with a Canon EOS REBEL T7 camera (Canon Inc., Tokyo, Japan) and with an optical microscope (SZ2-ILST, Olympus, Tokyo, Japan) coupled to an Olympus E-330 camera. For quantification, mineralized nodules were solubilized with 10% cetylpyridinium chloride (500 µL/well) under agitation (3 min), and absorbance was read at 570 nm. Results were shown as percentages relative to the osteogenic control (100%). The assay was performed in quadruplicate and repeated twice.

### RT-qPCR

The hDPCs were seeded at 2.5×10⁵ cells/well for 3-day assays and 5 × 10⁴ cells/well for 7- and 14-day assays in 6-well plates. They were exposed to MaquiCPI-3 at 0.05 µg/µL and the control (α-MEM + 10% FBS with osteogenic factors). Cells were collected with 10 µL β-mercaptoethanol (Sigma-Aldrich) in 1 mL lysis buffer from the PureLink™ RNA Mini Kit (Invitrogen, Carlsbad, CA, USA). RNA was extracted using the kit according to the manufacturer’s instructions. RNA concentration and quality were assessed with a spectrophotometer (NanoDrop™ 1000, Thermo Scientific, Wilmington, DE, USA). CDNA was synthesized using the High-Capacity cDNA Reverse Transcription Kit (Applied Biosystems, Life Technologies, Foster City, CA, USA). Quantitative real-time PCR (qPCR) was conducted using TaqMan Gene Expression Assays (Applied Biosystems, Life Technologies) with 1 µL of cDNA (25 ng/µL). The target genes included bone morphogenetic protein-2 – (Hs00154192_m1), osteocalcin – OC (Hs01587814_g1), alkaline phosphatase – ALP (Hs01029144_m1), bone sialoprotein – BSP (Bt03212717_g1), runt-related transcription factor 2 – RUNX2 (Hs01047973_m1), collagen type I alpha 1 – COL1A1 (Hs00164004_m1), dentin matrix protein 1 – (Hs01009391_g1), and dentin sialophosphoprotein – DSPP (Hs00171962_m1). Gene expression was normalized to the endogenous control glyceraldehyde-3-phosphate dehydrogenase (Hs02758991_g1). All assays were purchased from Applied Biosystems. Transcripts were analyzed using the StepOne™ Sequence Detection System (Applied Biosystems). Relative mRNA expression was calculated using the ΔΔCt method (fold expression = 2⁻(ΔΔCt ± stdev)). For dentin matrix protein 1 and DSPP, RT-qPCR was performed with two cDNA concentrations (25 ng/µL and 50 ng/µL).

### Cell Proliferation

Cell proliferation was evaluated by bromodeoxyuridine (BrdU) incorporation using an ELISA kit (Roche GmbH, Heidelberg, Germany). The hDPCs were seeded (1.3×10⁴ cells/well) in 96-well plates and after 24 h were treated with MaquiCPI-3 at 0.025, 0.05, and 0.1 µg/µL or controls (incomplete α-MEM and α-MEM + 10% FBS) for 24 and 48 h. BrdU was added after 6 h (24-h group) or 30 h (48-h group) of exposure, and the cells were incubated for 18 h at 37°C according to the manufacturer’s instructions. They were then fixed, incubated with anti-BrdU antibody, washed, and exposed to the substrate solution. Absorbance was measured at 370 nm (Asys UVM 340, Cambridge, UK), and results were shown as percentages relative to the FBS control. The experiments were performed in sextuplicate and repeated twice.

### Chemotaxis

A Transwell assay was performed to evaluate the chemotactic potential of MaquiCPI-3. The hDPCs (3×10⁴ cells/well) were seeded in the upper chamber of Transwell inserts (Corning^®^ FluoroBlok™ Cell Culture Insert, 8.0 µm pore size, Corning, USA) with 200 µL of α-MEM + 1% FBS. After 1 h, the lower chamber was filled with 600 µL of α-MEM + 1% FBS containing MaquiCPI-3 at 0.05 µg/µL or controls (α-MEM + 1% FBS and α-MEM + 10% FBS), the latter serving as the positive control. In total, two Transwells were used per group. After 24 h at 37 °C and 5% CO₂, the media were removed and the cells were washed with PBS, fixed with 4% paraformaldehyde, and stained with 4’,6-diamidino-2-phenylindole (DAPI, 1 μg/mL) (Life Technologies, Carlsbad, CA, USA). Overall, five random fields per Transwell were imaged using a fluorescence microscope (EVOS FL, AMC, Bothell, WA, USA) at 4× magnification. Nuclei were counted on ImageJ (National Institutes of Health – NIH, Bethesda, MD, USA).

### Statistical analysis

Data were analyzed on GraphPad Prism 9.0.2 (GraphPad Software, La Jolla, CA, USA) at a 5% significance level. Data normality was assessed by the Shapiro–Wilk or D’Agostino–Pearson tests and variance homogeneity by the Bartlett’s test. For normally distributed and homogeneous data, one-way ANOVA followed by Tukey’s post hoc test were applied (MTT, proliferation, chemotaxis). For non-normally distributed data, the Kruskal–Wallis test with Dunn’s post hoc test was used (alizarin red). For normally distributed data with heterogeneous variances, Brown–Forsythe and Welch’s ANOVA with Dunnett’s post hoc test (ALP activity) or two-way ANOVA with Šídák’s test were employed (qPCR).

## Results

### Inhibition of human cathepsin K activity

**Figure 1:**
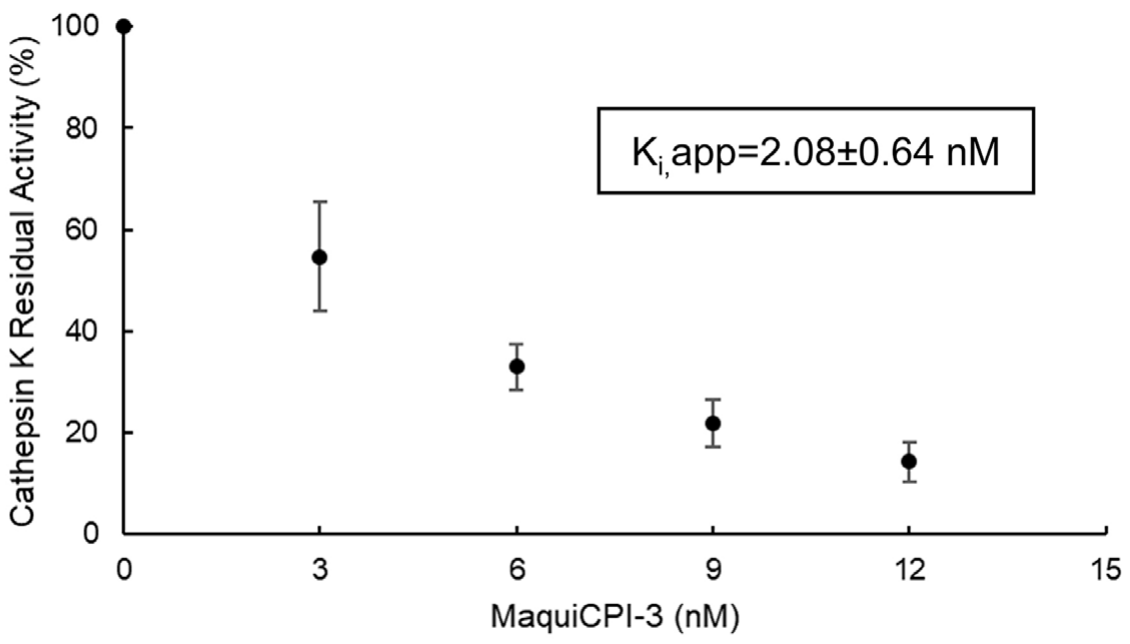
Inhibition curve of cathepsin K by MaquiCPI-3. Cathepsin K was activated in 100 mM sodium acetate buffer (pH 5.5) with 2.5 mM DTT for 5 minutes. The fluorogenic substrate (Z-Phe-Arg-AMC) was added, and fluorescence was monitored for 60 s. The inhibitor was added in increments, and fluorescence was measured after each addition. The curve shows residual enzymatic activity (initial velocity divided by velocity after inhibitor addition) versus increasing concentrations of the inhibitor. The residual activities were used to assess the apparent inhibition constant (Ki,app). Data are shown as mean ± standard deviation from three independent experiments.

The residual enzymatic activity following incremental concentrations of the inhibitor is shown in Figure 1. Graphical analysis evinced that MaquiCPI-3 had a K_i_ value of 1.72±0.57 nM and K_i_,app = 2.08±0.64 nM.

### Phenotypic characterization of hDPC cultures

**Figure 2:**
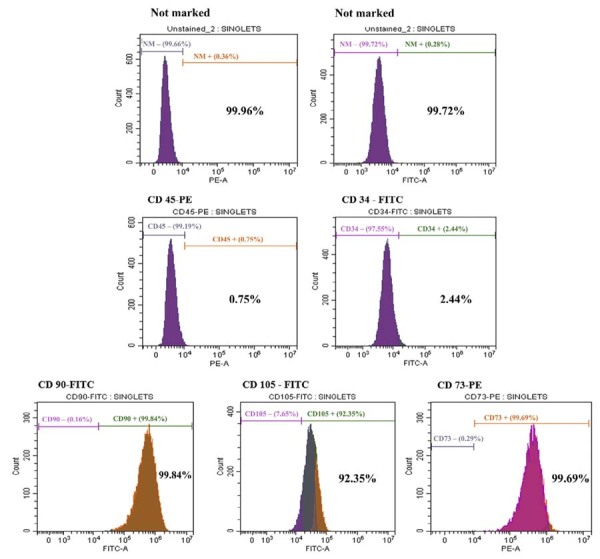
Characterization of hDPCs by flow cytometry. Representative histograms of unstained cells (PE and FITC channels), MSC marker expression (CD90, CD105, and CD73), and hematopoietic markers (CD45 and CD34).

The hDPCs expressed high levels of MSC markers, including CD90 (99.84%), CD105 (92.35%), and CD73 (99.69%), whereas hematopoietic markers CD45 (0.75%) and CD34 (2.44%) were detected at low percentages (Figure 2).

### Cell viability/metabolic activity assessed by MTT assay

**Figure 3:**
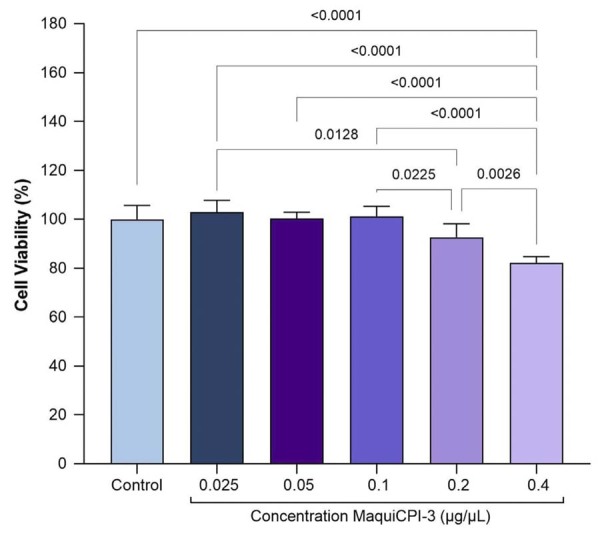
MTT assay. Viability of human dental pulp cells after exposure to concentrations of MaquiCPI-3 for 24 hours when compared with α-MEM + 10% FBS (control).

The results (Figure 3) showed no statistically significant differences in the viability of control cells when compared with those exposed to MaquiCPI-3 at concentrations of 0.025, 0.05, 0.1, and 0.2 µg/µL (*p*>0.05). At 0.4 µg/µL, MaquiCPI-3 reduced viability when compared with the control (*p*<0.05). However, values remained above 80%, which the International Organization for Standardization 10993-5:2009 (R2014) deems as non-cytotoxic. Based on these results, MaquiCPI-3 concentrations of 0.025, 0.05, and 0.1 µg/µL were selected for subsequent assays.

### Alkaline phosphatase (ALP) activity

**Figure 4:**
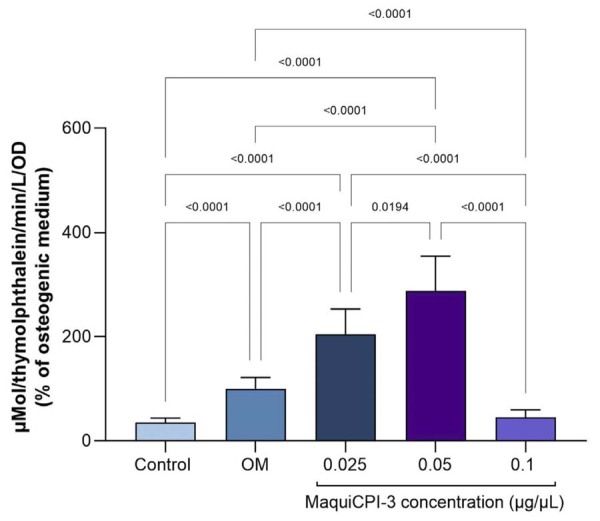
Alkaline phosphatase activity. Enzymatic activity after exposure to concentrations of MaquiCPI-3 for 14 days. OM, osteogenic medium.

MaquiCPI-3 at concentrations of 0.025 and 0.05 µg/µL induced ALP activity, with values significantly higher than those in the osteogenic medium (*p* < 0.05). At 0.1 µg/µL, ALP activity was lower than that of the osteogenic control (*p*<0.05) (Figure 4).

### Alizarin red staining (ARS)

**Figure 5:**
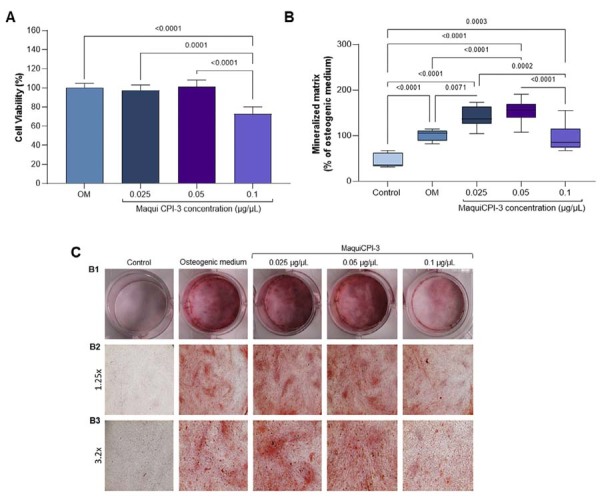
MTT assay and Alizarin Red staining. (A) Viability of human dental pulp cells after exposure to concentrations of MaquiCPI-3 for 17 days when compared with the osteogenic medium (control). (B) Comparison of mineralized nodule formation after 17 days of treatment with MaquiCPI-3 and controls, expressed relative to the osteogenic group. (C1) Images of 24-well plates obtained with a digital camera, and optical microscopy images at original magnifications of 1.25 (C2) and 3.2× (C3), showing mineralized nodules.

After 17 days, the MTT assay showed that MaquiCPI-3 at the lower concentrations (0.025 and 0.05 µg/µL) caused no cytotoxicity to hDPCs since no statistically significant differences were observed when compared with the control (*p*>0.05). At the highest concentration (0.1 µg/µL), cell viability decreased (72.95%) when compared with the control (*p*<0.05). A greater formation of mineralized nodules was observed in the osteogenic medium than in the non-osteogenic medium (*p*<0.05), confirming the effectiveness of the osteogenic components. MaquiCPI-3 at 0.025 and 0.05 µg/µL induced greater formation of mineralized nodules than the osteogenic medium (*p*<0.05) (Figure 5).

### RT-qPCR

**Figure 6:**
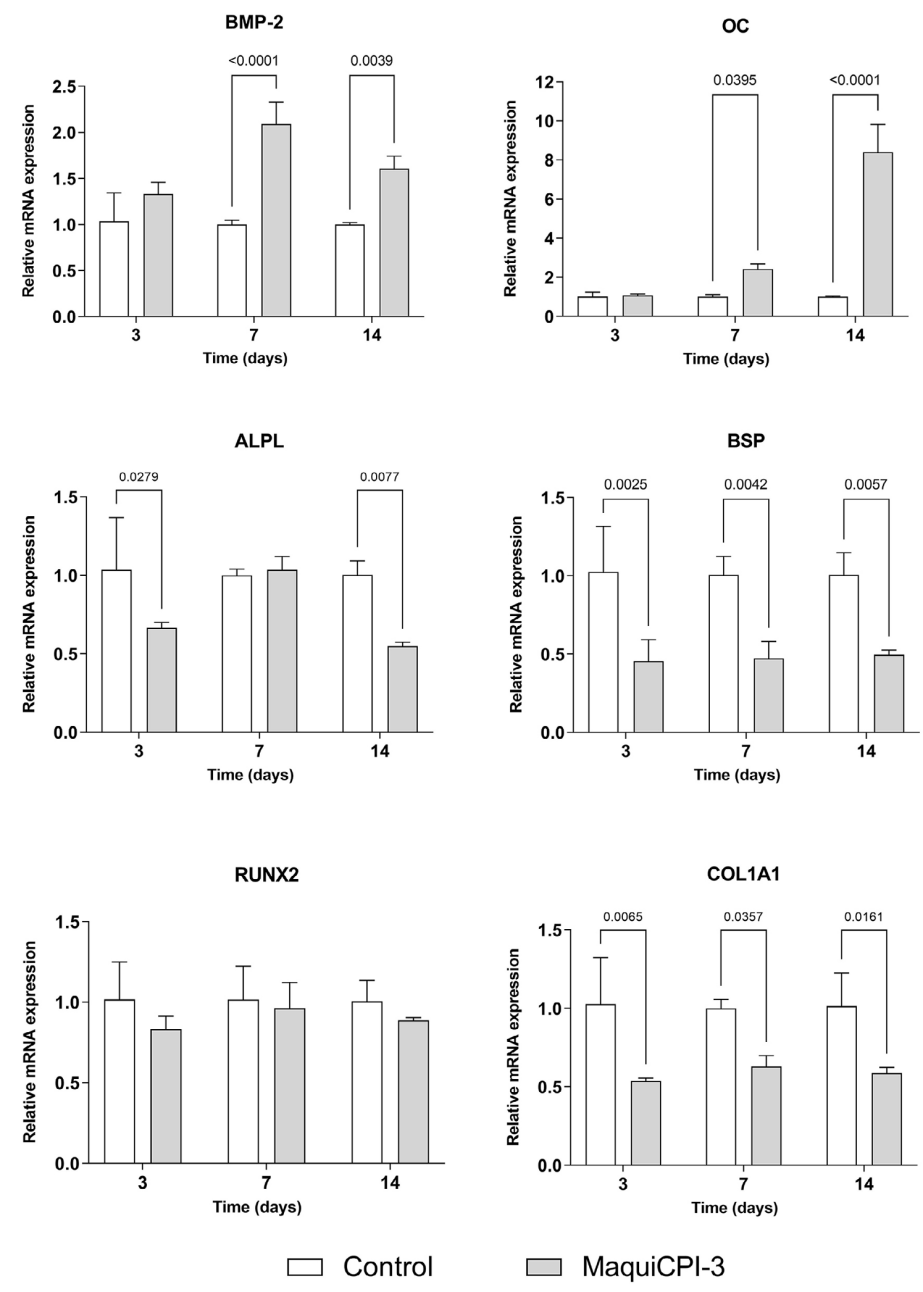
RT-qPCR assay. Relative mRNA expression levels of BMP-2 (A), OC (B), ALP (C), BSP (D), RUNX2 (E), and COL1A1 (F) in hDPCs exposed to 0.05 µg/µL of MaquiCPI-3 and control (α-MEM + 10% FBS with osteogenic supplements) for 3, 7, and 14 days using 25 ng/µL of cDNA.

Upregulation of BMP-2 and OC was observed in the MaquiCPI-3 group when compared with the control at 7 and 14 days (*p*<0.05). RUNX2 expression failed to differ significantly between groups (*p*>0.05), whereas ALP, BSP, and COL1A1 were downregulated in the treated group when compared with the control (*p*<0.05) (Figure 6). Transcripts of DMP1 and DSPP were not detected.

### Cell proliferation

**Figure 7:**
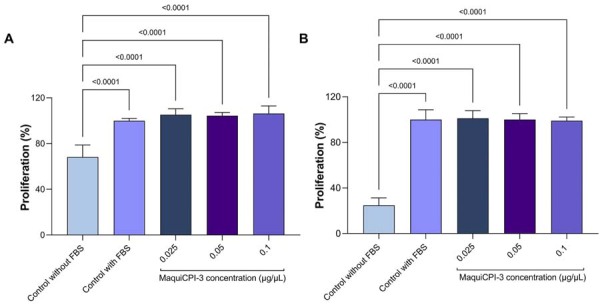
Cell proliferation assay (BrdU incorporation). HDPCs were cultured in control groups without fetal bovine serum (FBS) and with FBS and with MaquiCPI-3 at 0.025, 0.05, and 0.1 µg/µL in FBS-supplemented medium for 24 (A) and 48 h (B).

The BrdU incorporation assay showed a significant difference between the control group without FBS and the control group with FBS (*p*<0.05). However, no significant differences were observed between the MaquiCPI-3 groups and the FBS control group at either 24 or 48 h (*p*>0.05), as shown in Figure 7.

### Chemotaxis

**Figure 8:**
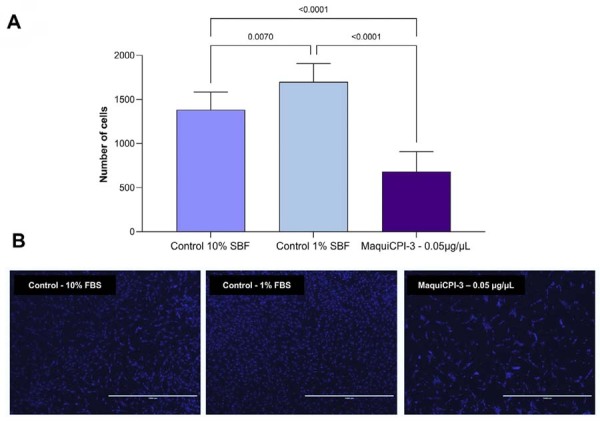
Chemotaxis assay. (A) Migration of hDPCs after exposure to MaquiCPI-3 at 0.05 µg/µL for 24 h when compared with α-MEM supplemented with 10% or 1% FBS (controls) using the Transwell assay. (B) Representative images of hDPC migration in each group. Cell nuclei were stained with 4’,6-diamidino-2-phenylindole (DAPI). Scale bar: 1000 μm (B).

The Transwell migration assay showed that MaquiCPI-3 at 0.05 µg/µL induced no chemotaxis since cell migration was lower than in the controls (*p*>0.05) (Figure 8).

## Discussion

This study evaluated the effect of MaquiCPI-3 on CTSK activity, cell viability, ALP activity, mineralized nodule formation, proliferation, chemotaxis, and the expression of osteogenic differentiation–related genes in hDPCs. The concentrations of 0.025, 0.05, 0.1, 0.2, and 0.4 µg/µL of MaquiCPI-3 were selected for the cytotoxicity assay based on a previous study using CaneCPI-5 in hDPCs^25^ and another study investigating MaquiCPI-3 for dental erosion control.^18^

Because of the adverse effects associated with synthetic CTSK inhibitors,^10^ safer alternatives have been sought, with cystatins—natural endogenous inhibitors of cysteine proteases—emerging as promising candidates.^26^ Recombinant phytocystatins act as inhibitors of human cathepsins, including CTSK,^14^ and have shown the ability to suppress osteoclastogenesis, attenuate inflammatory processes, and reduce bone loss related to periodontal disease.^12^ MaquiCPI-3 showed strong affinity for human CTSK, with a K_i_ of 1.72±0.57 nM. Direct measurement of Kᵢ is recognized as the most reliable method for assessing cystatin-mediated CTSK inhibition. Such a low K_i_ classifies this phytocystatin as a tight-binding inhibitor, i.e., a compound that binds to the enzyme with an apparent inhibition constant equal to or less than the total enzyme concentration in the assay.^27^ This is the first study to report CTSK inhibition by MaquiCPI-3, underscoring its potential as an adjuvant in endodontic therapy.

Human dental pulp represents a well-established source of MSCs,^28^ making it an appropriate experimental model to investigate the potential of bioactive compounds to induce osteogenic and odontogenic differentiation.^24^ Flow cytometry confirmed that more than 90% of the cultured cells expressed typical MSC markers, whereas fewer than 2.5% expressed hematopoietic lineage markers.^24^

HDPCs were used in this study as a representative model of dental mesenchymal stem cells. HDPCs and SCAPs share similar phenotypic characteristics and differentiation potential,^28^ and hDPCs have been consistently used to investigate biomaterials and bioactive compounds in the pulp–dentin–periapical complex repair.^25^

Pulpal and periapical repair involves the migration and proliferation of MSCs into the injured region, followed by their differentiation into osteoblast- and odontoblast-like cells, ultimately forming mineralized tissue.^29^ Therefore, materials used in endodontic therapy should support these cellular processes.^30^ MaquiCPI-3 induced cell migration/chemotaxis, which is consistent with previous findings for CaneCPI-5, another phytocystatin that showed no chemotactic effect, albeit evaluated by a different method (scratch assay).^13^ Similarly, MaquiCPI-3 neither stimulated nor impaired proliferation since its rates were comparable with those of the controls.

Although cathepsin K has been associated with cell migration and invasion in other cell types, particularly by its regulation of extracellular matrix turnover and chemokine processing,^31,32^ these effects highly depend on the cellular context. In hDPCs, migration is mainly regulated by chemokine-driven signaling, especially the SDF-1/CXCR4 axis and its downstream FAK/PI3K/Akt and GSK3β/β-catenin pathways^33^ rather than by cathepsin-mediated matrix remodeling. Under the non-inflammatory and non-osteoclastic conditions in this study, CTSK expression is expected to be low. Therefore, the absence of a chemotactic response to MaquiCPI-3 is consistent with the known biology of hDPCs.

Conversely, MaquiCPI-3 stimulated ALP activity in hDPCs. ALP is a key marker of early osteogenic and odontogenic differentiation, produced during the maturation stages of osteoblasts and odontoblasts. This enzyme plays a critical role in biomineralization and is widely used as an indicator of the bioactivity of mineralization-inducing compounds.^34^ The ability of MaquiCPI-3 to promote a mineralizing phenotype was further confirmed by its induction of mineralized nodule formation. The MTT assay, performed in parallel with the ARS experiment to monitor cell viability, confirmed that MaquiCPI-3 maintained hDPCs viability at the tested concentrations, supporting the reliability of the mineralization result, which are consistent with previous studies showing that recombinant cystatins from sugarcane and sweet orange promote osteogenic differentiation in human dental pulp cells and pre-osteoblasts.^13-15^

At the molecular level, MaquiCPI-3 increased BMP-2 and OC expression at 7 and 14 days. BMP-2 is a potent osteogenic inducer that promotes osteoblast differentiation and bone formation.^35^ OC is a non-collagenous bone matrix protein and a late marker of osteoblast differentiation.^36^ These findings are comparable with results in bone marrow cells treated with cystatin C, in which BMP-2 expression was observed at 7 and 21 days and OC expression at 7 and 14 days.^37^ Interestingly, BMP-2 is usually expressed at early stages of osteogenic and odontogenic differentiation,^14,37^ suggesting that MaquiCPI-3 may modulate BMP-2 expression at later stages. This expression profile also agrees with earlier findings showing progressive OC upregulation from days 0 to 15 in MSCs from dental pulp and apical papilla cultured in osteogenic medium.^38^

RUNX2 expression showed no difference from controls, whereas ALP, BSP, and COL1A1 were downregulated in the MaquiCPI-3 group. RUNX2 is a transcription factor that coordinates multiple signaling pathways during osteoblast differentiation.^37^ BSP, mainly expressed by osteoblasts, is associated with matrix deposition and mineralization.^39,40^ ALP is a key early osteogenic/odontogenic marker that plays an important role in bone mineralization.^41^ Evidence suggests that osteogenic differentiation and bone metabolism may be partly regulated by microRNAs.^42^ Specific microRNAs may have downregulated BSP and ALP expression in hDPCs, even in the presence of other osteogenic markers.

Transcripts of the odontoblastic markers DMP1 and DSPP were not detected. DMP1 is secreted into the predentin and dentin extracellular matrix, regulating hydroxyapatite nucleation and growth. DSPP, a precursor of bioactive dentin matrix proteins, plays a central role in dentin mineralization.^43^ Notably, RT-qPCR assays with two cDNA concentrations (25 and 50 ng/µL) yielded detectable expression of these genes.

Mechanistically, the pro-osteogenic effects of MaquiCPI-3 are consistent with its inhibition of CTSK (K_i_app = 2.08±0.64 nM). CTSK blockade may reduce extracellular matrix degradation and attenuate pro-inflammatory cytokine production,^2^ helping to reestablish the balance between bone resorption and formation. This more stable and less inflammatory microenvironment favors the osteogenic behavior of dental mesenchymal stem cells and stimulates the expression of genes associated with differentiation and mineralization.^44^ In parallel, evidence from other recombinant phytocystatins indicates that these cathepsin K inhibitors can directly modulate the intracellular pathways in osteogenesis: C*sin*CPI-2 increased BMP-2, RUNX2, ALP, osteocalcin, BSP,^14^ and mineralized nodule formation in dental-derived cells.^14,25^ In this study, MaquiCPI-3 similarly upregulated BMP-2 and osteocalcin expression and enhanced ALP activity and mineralized nodule formation.

Considering its ability to inhibit CTSK activity and promote osteogenic phenotype, MaquiCPI-3 represents a promising molecule for direct pulp protection and regenerative endodontic procedures aimed at pulp–dentin–periapical complex repair. In apical periodontitis, MaquiCPI-3 may help control bone resorption by inhibiting CTSK and promoting repair via osteogenic differentiation.

Despite these promising findings, limitations of the *in vitro* model must be acknowledged since it fails to fully reproduce *in vivo* cellular and molecular interactions. Further studies are required to evaluate MaquiCPI-3, including quantification of its cathepsin K–inhibitory activity under inflammatory cell-culture conditions or in osteoclast cultures, and to relate these findings to osteogenic differentiation and its assessment *in vivo* models. Moreover, its incorporation into vehicles or biomaterials should be investigated to enable clinical translation for dentin–pulp–periapical tissue repair.

## Conclusion

MaquiCPI-3 inhibited human CTSK activity, showed no cytotoxicity, and induced osteogenic differentiation of hDPCs. Although it stimulated no cell proliferation or migration, its ability to promote an osteogenic phenotype and inhibit CTSK highlights its potential as a therapeutic strategy for pulp and periapical repair and for bone resorption control.
